# Influenza-Like Illness Surveillance on Twitter through Automated Learning of Naïve Language

**DOI:** 10.1371/journal.pone.0082489

**Published:** 2013-12-04

**Authors:** Francesco Gesualdo, Giovanni Stilo, Eleonora Agricola, Michaela V. Gonfiantini, Elisabetta Pandolfi, Paola Velardi, Alberto E. Tozzi

**Affiliations:** 1 Multifactorial Diseases and Complex Phenotypes Research Area, Bambino Gesù Children’s Hospital IRCCS, Rome, Italy; 2 Department of Informatics, “Sapienza” University of Rome, Rome, Italy; National University of Singapore, Singapore

## Abstract

Twitter has the potential to be a timely and cost-effective source of data for syndromic surveillance. When speaking of an illness, Twitter users often report a combination of symptoms, rather than a suspected or final diagnosis, using naïve, everyday language. We developed a minimally trained algorithm that exploits the abundance of health-related web pages to identify all jargon expressions related to a specific technical term. We then translated an influenza case definition into a Boolean query, each symptom being described by a technical term and all related jargon expressions, as identified by the algorithm. Subsequently, we monitored all tweets that reported a combination of symptoms satisfying the case definition query. In order to geolocalize messages, we defined 3 localization strategies based on codes associated with each tweet. We found a high correlation coefficient between the trend of our influenza-positive tweets and ILI trends identified by US traditional surveillance systems.

## Introduction

Digital traces left on the Internet by web users, if properly aggregated and analysed, hold the promise to inform syndromic surveillance systems with real time data collected directly from individuals [1].

A number of studies have focused on measuring the occurrence of specific health-related and disease-related search keywords. In some cases, a correlation between search volumes and disease trends has been identified [2] and, in 2008, a Google service has been developed to estimate and predict influenza activity by aggregating Google search query volumes [3,4]. Nevertheless, this demand-based approach can suffer from a high level of “noise”: indeed, web users search for health subjects of which they have close experience, but often search peaks can be completely unrelated to the incidence of a disease, as search behaviors change in time and discussions on traditional media may reflect on search patterns [5,6].

“Supply-based” infodemiology on the other hand, aims straight at what web users speak about, investigating communication contents and patterns in discussion groups, blogs and microblogs [7]. In such environments, keywords occur in contexts, which allow the use of text mining techniques for sense disambiguation, topic filtering and mood analysis [8,9]

Twitter, a popular free networking and microblogging service, counting in 2012 500 million users generating over 300 million tweets daily [10], has also been analysed as a source of syndromic surveillance data [11,12]. One of the strong implications of the use of Twitter for infodemiology is that it provides location indicators [13], potentially allowing a constant, dynamic and real-time update of disease maps [14].

Previous studies for tweet-mining measured the occurrence of single pre-specified terms, consisting either in the name of a clinical condition or its synonyms (eg: H1N1 or swine flu) [11] or in words, arbitrarily chosen by the authors, related to the clinical syndrome itself (eg. flu, vaccine, tamiflu) [12].

This kind of approach may suffer from two major biases. 

First, in blogs and forums, people are motivated by a communication need (possibly among pairs), rather than by an information need and therefore naïve language is often preferred to technical language. 

Secondly, it is likely that, in their tweets, most users will describe a combination of symptoms rather than a diagnosis. An approach that takes into account only disease-related keywords can miss a large volume of messages in which users include a mix of signs and symptoms that can actually describe a clinical syndrome. 

In order to address these biases, we first developed a minimally supervised algorithm to learn technical term-naïve term pairs, based on pattern generalization and complete linkage clustering, and we applied it to a group of technical terms extracted from the European Centre for Disease Prevention and Control (ECDC) case definition for ILI. Subsequently, we built a Boolean query based on the ECDC case definition for ILI, using both technical and related jargon terms as identified by the algorithm. Using the available APIs, we collected two sets of Twitter messages matching the ILI query, and we compared the trends of these messages with traditional surveillance data for influenza in the US.

## Materials and Methods

### Algorithm development: extraction of naïve-medical jargon

We developed an algorithm that automatically maps all naïve terms related to a specific medical term from Freebase (www.freebase.com/view/medicine/disease), exploiting the abundance of web pages that aim at popularizing medical topics (e.g.: “chills are the frequent name for a feeling of coldness“, or “sore throat, your doctor would call it pharyngitis”). The algorithm starts with an initial small learning set of medical conditions, composed by term pairs (1 technical and 1 naïve term, e.g.: emesis-vomiting) to extract basic patterns from the web, and then generalize, cluster and weight these patterns based on another small set of pairs. Generalized patterns are learned both for sentence fragments that relate technical and naïve terms (e.g.: “a common term for” →“ #DT #JJterm *for*”), and for multi-word expressions describing medical conditions (e.g. “inflammation of the nose” → “inflammation of BODYPART”). Patterns are based on lexical, syntactic and semantic features. The performance of the algorithm is evaluated on a “golden” test set of pairs extracted from Freebase (www.freebase.com/view/medicine/disease), and through manual evaluation by domain experts.

For a complete description of the algorithm, see Information S1.

### Query development

In order to analyse the performance of Twitter as a source of data for syndromic surveillance, we developed a Boolean query derived from an ILI case definition. We first considered the translation of the ILI case definition adopted by the CDC [15]: fever and a cough and/or a sore throat without a known cause other than influenza. Nevertheless, translating this case definition into a Boolean query was not straightforward, as the generic expression “without a known cause other than influenza” cannot be transformed in an effective search string. Since the scope of our work was to test a tool for syndromic surveillance based on an aggregation of symptoms, we decided to adopt the ECDC case definition [16]: “Sudden onset of symptoms AND at least one of the following four systemic symptoms fever or feverishness, malaise, headache, myalgia AND at least one of the following three respiratory symptoms: cough, sore throat, shortness of breath”. This case definition includes more symptoms compared to the CDC ILI case definition and does not take into account any generic expressions (apart from the “sudden onset of symptoms” that we did not include in our query, as it was not easily translatable into a search term-string).

First, we applied our algorithm to a set of 8 symptom-related medical conditions expressed as technical terms derived from the above mentioned case definition, (e.g. *fever, feverishness, malaise, headache, myalgia, cough, pharyngitis, dyspnea*), obtaining an additional set of naïve terms, as described in Table 1.

**Table 1:** Terms extracted from ECDC influenza case definition and synonyms identified by the algorithm.

*Case Definition Term*	*Synonym clusters*
**fever**	fever, high temperature, pyrexia, febrile convulsion
**feverishness**	feverishness, chills, rigors, feeling of coldness, coldness, trembling, shivering
**malaise**	malaise, unease, discomfort, weakness, feeling of sickness, feel sick, bodily discomfort, body aches, body pain, pain in body
**headache**	cephalgia, cephalodynia, cephalea, head ache, headache, migraine, head pain, migraines, head hurts, headachey
**pharyngitis**	pharyngitis, sore throat, laryngitis, sore throat, bad throat, painful throat, scratchy throat, itchy throat, tonsillitis, raw throat, irritated throat, throat hurt, throat tickle, throat inflammation
**dyspnea**	shortness of breath, difficult breathing, breathlessness, troubled breathing, air hunger, congested chest, can’t breath
**myalgia**	myalgia, muscular pain, muscle ache, muscle pain, painful spasm
**cough**	cough, coughing

Secondarily, we transformed the ECDC ILI case definition into a Boolean query using both the original technical terms and the related jargon terms as identified by the algorithm: (*(fever*)*OR(feverishness*)*OR(malaise*)*OR(headache*)*OR(myalgia*))*AND*

*((cough*)*OR(pharyngitis*)*OR(dyspnea*))

where we omitted to expand query terms with the “OR” of their alternative terms for brevity. 

### Twitter data

We analysed Twitter data on two different datasets.

The first dataset (Dataset 1) was derived from a 1% sample of the worldwide Twitter traffic acquired between November 11^th^, 2012 and April 27^th^, 2013 through the available application programmer’s interfaces (APIs, https://dev.twitter.com/docs/streaming-apis).

As for the second dataset (Dataset 2), starting from January 27^th^, 2013 and until May 2013, , we collected a set of Twitter messages including at least one of the singleton terms composing the ILI query and 3 additional queries based on other case definitions (Cold, Gastroenteritis, Allergy) adopted by the Influenzanet system [17]. The analysis of the tweets satisfying the non-ILI case definitions is not included in this report. A total of 17 technical keywords + 65 jargon keywords were included. This allowed us to retrieve almost 100% of the total Twitter traffic with those terms.

We built a system that allows monitoring collected tweets and produce time series for the requested information, similarly to Google Trends (http://www.google.com/trends/). Contrary to Google Trends, our system shows the absolute frequency of terms and allows for complex Boolean queries. 

### Geolocalization

In order to identify tweets localized in the US, we first took into account only those tweets sent by mobile phones reporting Global Positioning System (GPS) coordinates. Secondly, we created 2 sets of extended criteria based on other location data (place code, time zone, place indicated in user’s profile) that can be extracted from each message. 

Summarizing, we obtained 3 groups of US-geolocalized tweets, each group being based on a different set of criteria:

1 US-GEO: tweets providing US GPS coordinates
2 US-WIDE: tweets responding to one of the following criteria:
  ◦ US GPS coordinates
  ◦ explicit US place code
  ◦ US related time zone
  ◦ place indicated in user’s profile included in those reported in http://en.wikipedia.org/wiki/List_of_U.S._states in the following fields: Common name, State Capital, Most populous city.
3 US-NARROW: same as previous set of criteria but excluding all tweets reporting a US time zone but a non-US place-code.

### Query evaluation

From our second dataset we extracted 100 tweets matching the query on influenza like illness, and a random sample of 500 tweets not matching the query, but including at least one symptom. Tweets were then examined independently by the three of the Authors (EA, FG, AET), to test consistency of extracted tweets with case definition. The tweet examination yielded a 3% false positive rate with a precision of 0.97. We have not found false negatives in the sample reviewed for assessing precision.

### Influenza-like illness data

Data for US ILI trends were those reported by the U.S. Outpatient Influenza-like Illness Surveillance Network (ILINet), collected through the Centers for Disease Control and Prevention (CDC) Fluview website (http://gis.cdc.gov/grasp/fluview/fluportaldashboard.html). The members of ILINet weekly report to the CDC the number of patient visits for influenza-like illness (ILI) by age group. In the US surveillance system a week begins with a Sunday, and ILI is defined as fever (temperature of 100°F [37.8°C] or greater) and a cough and/or a sore throat without a known cause other than influenza.

### Control series

As suggested by Bodnar and Salathé [18], some models built on Twitter series can fit the data even using ILI non-related keywords. Despite we did not build any model for influenza prediction, we decided to use a series of tweets containing ILI non-related keywords in order to measure its correlation with ILINet data. We chose the same keywords used by the authors [18], namely "zombie" OR "zed" OR "undead" OR "living dead", and compared our non-ILI trend with ILINet data. 

### Statistical analysis

Results of tweet trends are reported as number of ILI positive tweets (or number of ILI negative tweets for the control series) in the unit of time (week).

In order to compare tweet trends with traditional surveillance trends, tweet data were aggregated considering a week starting from a Sunday. Results of tweet trends, ILINet data and Google Flutrends data are expressed as z-scores. 

Pearson correlation coefficients were used to compare US surveillance data with Twitter traffic consistent with the ILI case definition and with Twitter traffic not consistent with the ILI case definition (non-ILI tweets). Twitter traffic was expressed as: total available tweet traffic, US-GEO tweets, US-WIDE tweets, US-NARROW tweets,. Total available traffic series for the 1% sample dataset and US-NARROW series for the second dataset were smoothed by Loess function [19].

Tweets from the 1% sample and US-NARROW tweets consistent with the ILI case definition were also compared with Google Trends data and with trends generated by tweets reporting the words “flu” OR “influenza”, geolocalized with the same strategy where appropriate.

## Results

### Dataset 1

The tweets corresponding to the 1% sample of the total worldwide Twitter traffic from November 11^th^ to April 27^th^ were 447,597,718. Of these, a total of 5,508 tweets satisfied the conditions set by the query for ILI. The sample of ILI tweets responding to the geo-localization criteria was too small, therefore we only took into account the total ILI tweet series in the analysis, smoothed by Loess function. Twitter and traditional surveillance trends for US were compared, and the correlation coefficient was high (0.981, p<0.001), higher than the correlation of ILINet data with Google Flu Trends (0.966, p<0.001) and with tweets containing the words “flu” or “influenza (0.899, p<0.001).

**Figure 1:**
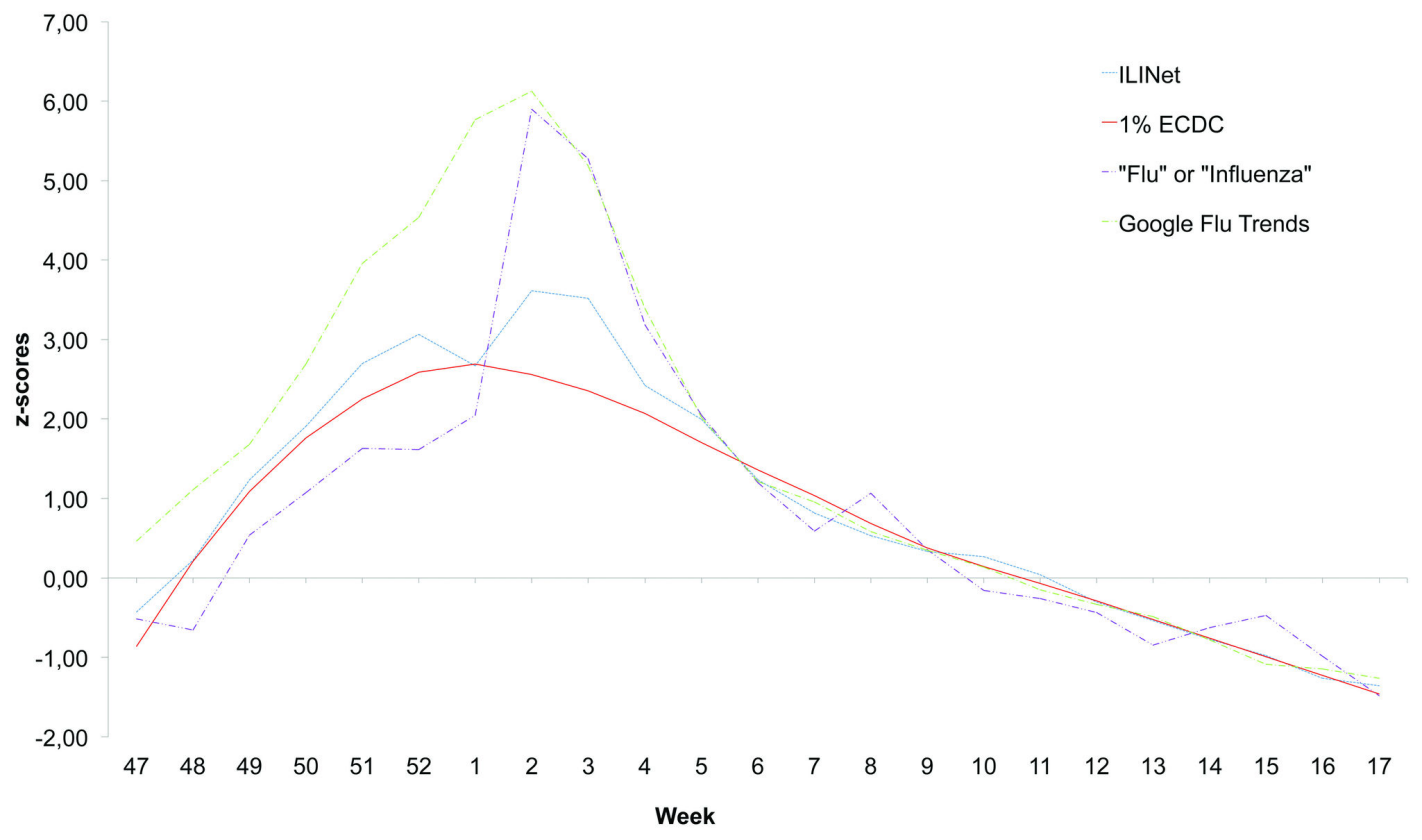
Weekly reported ILI (CDC) and tweets satisfying ILI query. The blue line represents the z-scores of CDC’s reported ILI for the 23-week period starting in week 47 (November 2012) through week 17 (May 2013). The red line represents the z-scores of tweets satisfying the ECDC ILI query. The purple line represents the z-scores of tweets including the words “flu” or “influenza”. The green line represents the z-scores of Google Flu Trends data.

Figure 1 shows the comparison between weekly ILI tweets (smoothed by Loess function), ILINet data, Google Flutrends and tweets containing the words “flu” or “influenza”. The figure shows that the series of tweets consistent with the influenza case definition does not overestimate the actual flu peak, as Google Flu Trends series and the series of tweets containing the words “flu” or “influenza” do.

We also compared ILINet data with the control series of tweets containing ILI non-related keywords, and, as expected, the correlation coefficient was very low (0.292, p=0.159).

### Dataset 2

As for the second database, from January 27^th^ to May 5^th^ 2013 we collected a total of 232,452,510 tweets containing at least one of the terms included in the ILI case definition and in the 3 additional Influenzanet case definitions, as mentioned above in the Twitter data section (Cold, Allergy, Gastroenteritis).

A total of 3,252,013 (1,3%) tweets responded to the US-GEO criteria, 85,381,987 (36%) responded to the US-WIDE criteria, 11,040,587 (47%) responded to the US-NARROW criteria.

**Figure 2:**
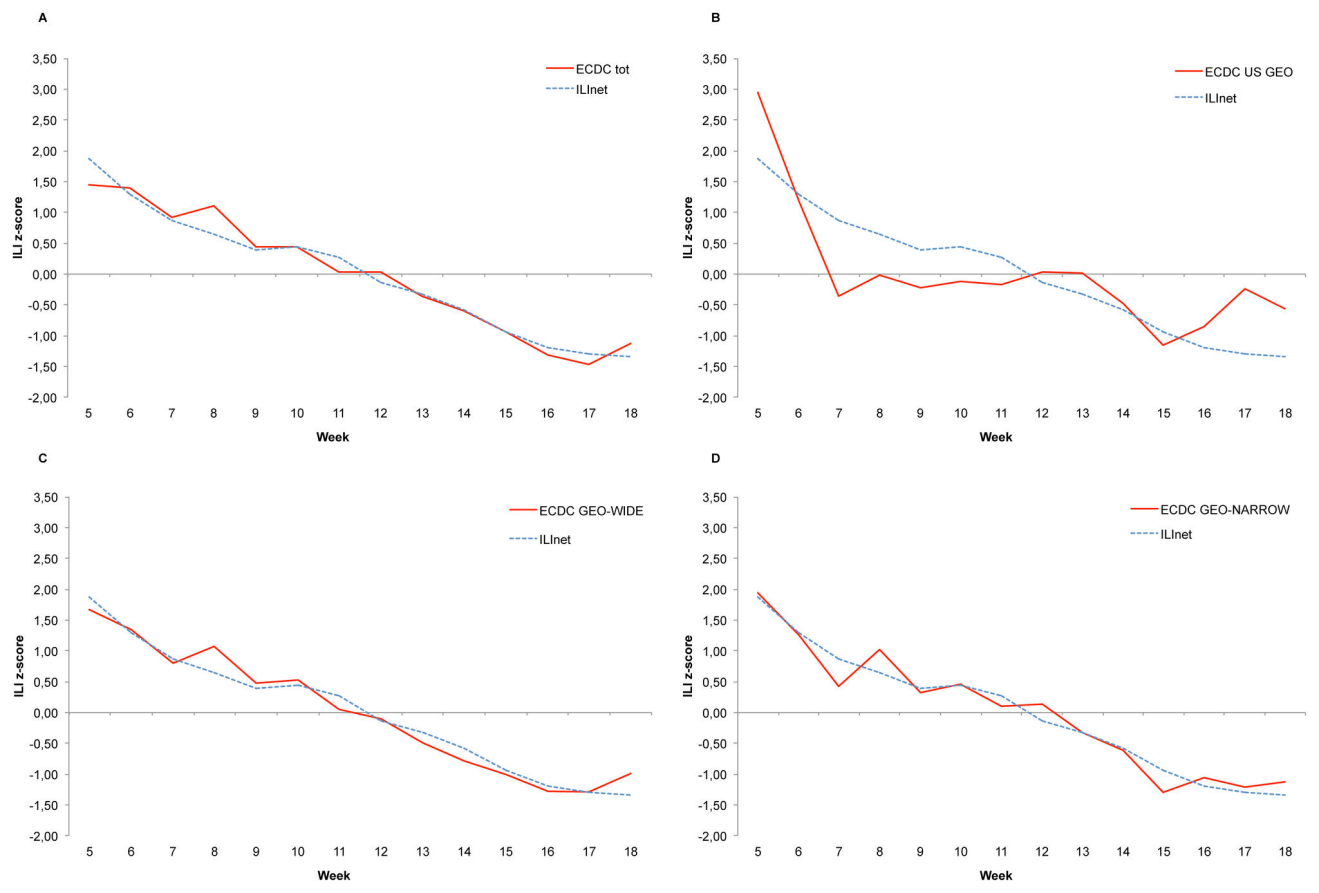
Weekly reported ILI (CDC) and tweets satisfying ILI query. The blue line represents in all graphs the z-scores of CDC’s reported ILI for the 14-week period starting in week 5 (January 2013) through week 18 (May 2013). The red line represents the z-scores of tweets satisfying the ECDC ILI query, selected with a different geolocalization strategy in each of the four graphs: a) all tweets (independently from geolocalization); b) US GEO(GPS localized tweets); c) Extended wide localization pattern; d) Extended narrow localization pattern.

Out of the total, 262,853 tweets (0.11%) satisfied the conditions set by the query for ILI. Comparison between ILI tweets and traditional surveillance trends for US is reported in Figure 2a-b-c-d. The highest correlation with ILINet data was observed for the US-WIDE tweet trend (Figure 2c, r=0.980, p<0.001), followed by all tweets independently from localization (Figure 2a, r=0.977, p<0.001), US-NARROW tweets (Figure 2d, r=0.974, p<0.001) and US-GEO tweets (Figure 2b, r=0.769, p=0.001). Comparison of ILINet data with US-NARROW tweets smoothed by Loess function yielded the highest correlation coefficient (r=0.997, p<0.001).

**Figure 3:**
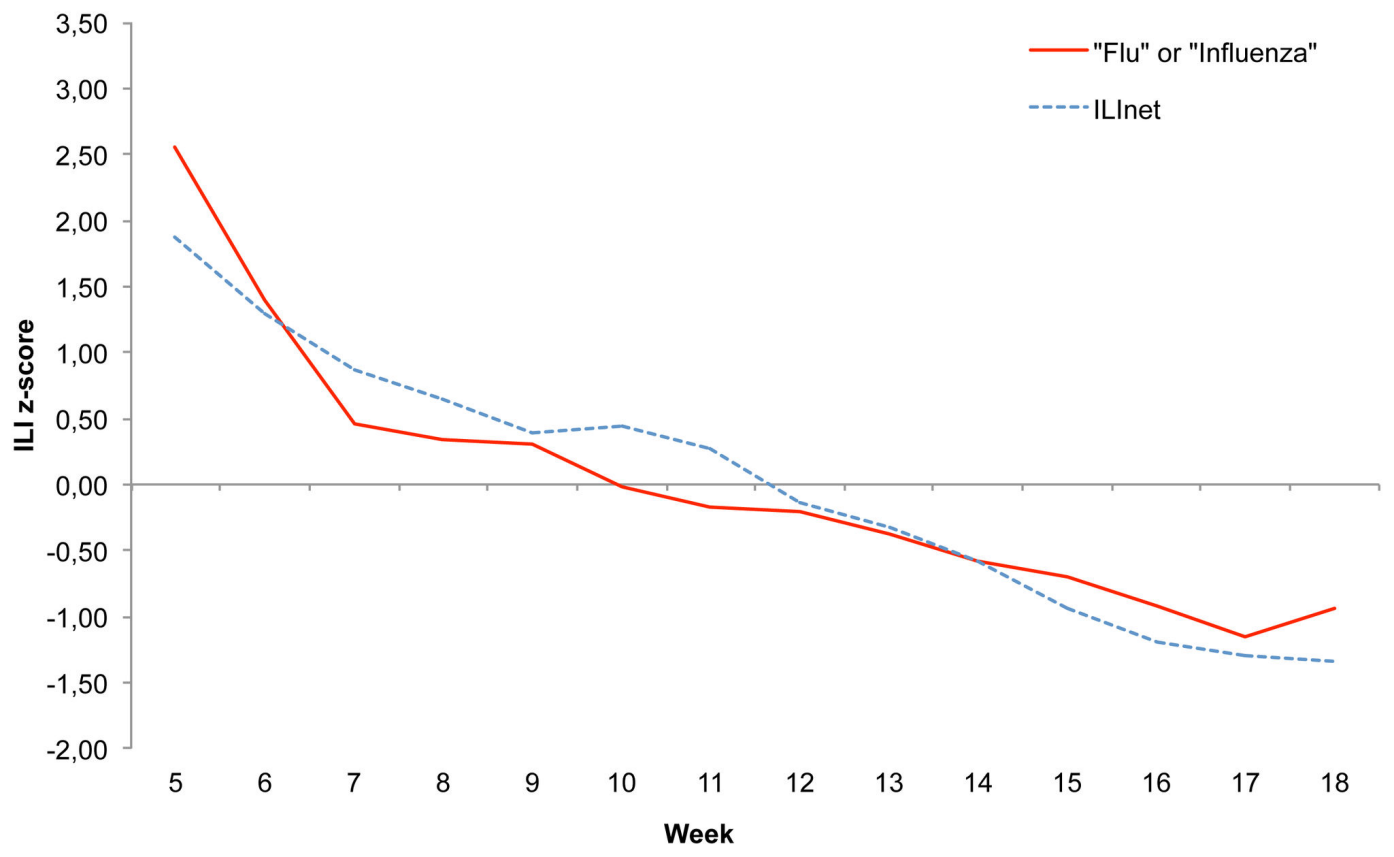
Weekly reported ILI (CDC) and tweets including the words “flu” or “influenza”. The blue line represents in all graphs the z-scores of CDC’s reported ILI for the 14-week period starting in week 5 (January 2013) through week 18 (May 2013). The red line represents the z-scores of tweets including the words “flu” or “influenza”, geolocalized with the extended narrow localization pattern.

Finally, when comparing ILINet data with tweets containing the word “flu” or the word “influenza”, geolocalized through the US-NARROW criteria, , we obtained a lower correlation coefficient compared to the tweet trend consistent with the ECDC case definition (r=0.944, p<0.001). Results are illustrated in Figure 3.

## Discussion

In the present study, we analysed Twitter messages as a source of data for syndromic surveillance, taking into account the use of non-medical language by microblog and social network users. First, we created an algorithm that automatically identifies jargon terms commonly used to express medical concepts in everyday language. Subsequently, we used a combination of symptoms, expressed both with technical and naïve terms, in order to identify tweet messages reporting a syndrome consistent with an ILI case definition. We found a very high correlation between tweet trends and traditional US surveillance data. 

The general public timely shares personal information on social networks and microblogs like Twitter, which can therefore be a precious source of data for disease surveillance and public health.

In particular, Twitter has previously been used for monitoring information sharing on cardiac arrest [20], dental pain [21], and behavior patterns [22]. In these studies, tweets were manually evaluated by a group of researchers for mainly descriptive purposes. 

Chew et al [11] performed content and sentiment analyses (both manually and automatedly) on a database of tweets containing the keywords “swine flu”, “swineflu” and/or “H1N1”, collected through an infoveillance system named Infovigil.

Signorini et al [23] used Support Vector Regression to estimate weekly ILI from usage statistics of a group of pandemic influenza-related terms that had been pre-specified by the authors. The words that were taken into account concerned the diagnosis itself (flu, swine, influenza, h1n1), therapies (tamiflu, oseltamivir, zanamivir, relenza, amantidine, rimantidine, vaccine), or were generic terms (symptom, syndrome, illness). 

Twitter-based surveillance, similarly to search-related surveillance, may be influenced by news and media reports, which have the potential to strongly affect the contents of tweets or google searches: Google Flu Trends (www.google.org/flutrends/‎) overestimated peak flu levels during the 2012-2013 flu season [6,24], and, interestingly, we found that a trend based on tweets including the word “flu” or “influenza” overestimated the flu peak in a similar way. 

Nevertheless, compared to search-related surveillance, Twitter mining has an interesting potential: the intrinsic nature of social networks (blogs, microblogs and forums), where words appear in specific contexts, allows a variety of natural language analyses and sense disambiguation techniques that may decrease the “noise” and increase our ability to detect true “signals” of disease.

We developed a Twitter monitoring instrument which detects the absolute frequency of indexed terms and performs complex Boolean queries, thus allowing to finely analyse presence and combinations of terms in tweets. We transformed the ECDC case definition for ILI into a Boolean query, in order to base our analysis on a combination of symptoms rather than on a suspected or final diagnosis. Through this approach we reduced the risk of including in the analysis tweets that mentioned the disease to express concern or fear (e.g.:“a little worried about flu epidemic!”) rather than reporting an infection [25]. We believe that this method has increased the specificity of our Twitter-mining process. As a matter of fact, the correlation of our tweet trend with ILINet data was higher compared with that of tweets only reporting the words “flu” or “influenza” and with Google Flu Trends for the same time period, indicating that our system is less affected by aspecific signals. The reliability of our system is also confirmed by the very low rate of false positives (3%) yielded by the manual examination of a sample of tweets: almost all ILI-tweets selected by our system truly reported a complaint of symptoms.

Another crucial novelty of our approach is an improvement in the sensitivity of the tweet mining by inclusion of jargon terms in our query. The algorithm we developed automatically learns a variety of expressions that people use to describe their health conditions, thus improving our ability to detect health-related “concepts” expressed in non-medical terms and, in the end, producing a larger body of evidence. For example, on February 3rd, in our dataset, *pharyngitis* cumulated 26 tweets, while their correspondent naïve terms occurred 234,951 times. 

We tested our system on a first dataset representing a 1% sample of the total tweet traffic circulating during the entire 2012-2013 influenza season. We compared tweets in English language consistent with the ECDC case definition with ILINet data, and we found a very high correlation coefficient (0.981). We can hypothesize that the majority of tweets in English language are sent from the US, and can therefore represent US trends with a good approximation. Despite the small sample and the lack of geolocalization, the high correlation shows that our approach is solid.

The analysis on the second dataset, although limited to the second phase of the influenza season, confirms the potential of our system for a more refined and precise syndromic surveillance. Tweets composing the second dataset included at least one health-related term extracted from a group of 5 different case definitions. This allowed us to perform our analysis on a much larger sample, thus decreasing variability and increasing precision. Moreover, the larger size of the sample allowed us to obtain geolocalized sub-samples of adequate size. 

We adopted 3 different geo-localization strategies in order to identify tweet trends localized in the US. In the first strategy, we selected only tweets that reported GPS coordinates. In the 2 “extended” strategies, we exploited other location data, including time zone, place code and place indicated in user’s profile. As opposed to the “narrow” extended localization pattern, the “wide” extended localization pattern did not exclude tweets that reported a discrepancy between time zone and place code. This original approach allowed us to identify a larger number of tweets than using GPS coordinates alone. 

Tweets localized with the “wide” extended strategy and reporting a combination of symptoms consistent with the case definition were those which better correlated with the ILINet trends. The correlation coefficient was very high (0.980). We also found a high correlation of all tweets (independently from geo-localization) with ILINet data (0.977). 

Overall, considering both databases, our best correlation coefficients are higher than those reported by others, either using Google Flu Trends [3,26] or Twitter [27]. 

Our study has several strengths. First of all, we used a flexible system that can be easily applied to different country settings and languages, provided a specific ontology exists, and to other kinds of syndromic surveillance, including surveillance of emerging diseases and allergies. Moreover, tweet analysis can explore the association between symptoms and specific exposures, with particular relevance to pharmacovigilance. The cost of our system is very low, and data can be acquired more quickly and timely compared to traditional surveillance systems.

Our study has also a number of limitations. First, Twitter users are not representative of the entire US population. As of February 2012, according to the Pew Internet Report, young adults, Afro-American and people living in urban areas are overrepresented on Twitter [28]. This may have biased our results, showing a trend that was specific for a restricted population group. Nevertheless, the correlation coefficient was quite high, and our system catches information on a segment of population that may not be entirely captured by traditional surveillance systems.

Secondarily, the case definition used by the CDC surveillance system for monitoring ILI in the US slightly differs from the one that we used for tweet mining, which was instead derived from the ECDC case definition. Although this may represent a potential bias, the correlation of our tweet trends and traditional surveillance trends was very high. The case definitions adopted by traditional surveillance systems are based on a clinical diagnosis made by a physician. Our study looks at the same issue from another perspective, exploring instead the way patients report their illness. We can speculate that, in this respect, the ECDC ILI case definition, compared to the CDC ILI case definition, is more likely to resemble a combination of complaints as reported by a patient. More research should focus on building case definitions that are specific for digital epidemiology.

Thirdly, the low rate of geo-localized tweets does not allow to draw definite conclusions of the reliability of our system for achieving a high spatial resolution of cases. Moreover, restricting the analysis to geo-localized tweets may also introduce a selection bias: it is possible that users who allowed GPS coordinates or included other localization codes in their profile differ from the rest of Twitter users. 

Moreover, the high temporal resolution of tweet data implies that the system is susceptible to daily and hourly fluctuations. Nevertheless the granularity of data allows to customize data in any time window relevant to surveillance, giving epidemiologic information more frequently, more timely and more rapidly than traditional surveillance systems. 

Finally, we tested our system on one influenza season only. Results need to be confirmed on next seasons.

## Conclusions

In conclusion, our study shows that Twitter-based infodemiology techniques can be improved by mining users’ messages through Boolean queries derived from disease case definitions, and by including jargon terms in the queries. This approach yields a high level of correlation with trends derived from traditional surveillance systems, thus being reliable and less sensitive to media reports compared to other digital epidemiology methods. Taking advantage of tweet geolocalization, it can provide quick and timely information for syndromic surveillance. Our method can also be applied to a variety of different case definitions, and to different country settings.

## Supporting Information

Supporting Information S1 **Extraction of naïve medical jargon: methods and results.**
(DOCX)
