# Supplementary material for: Influenza-Like Illness Surveillance on Twitter through Automated Learning of Naïve Language
Source: PLoS One. 2013 Dec 4;8(12):e82489. doi: 10.1371/journal.pone.0082489 (PMC3853203; doi:10.1371/journal.pone.0082489)
Supplement: Supporting Information S1 — Extraction of naïve medical jargon: methods and results. (DOCX) [file pone.0082489.s001.docx]

# EXTRACTION OF naïve MEDICAL JARGON: METHODS and RESULTS

This section briefly summarizes the algorithm to automatically map technical and naïve terms describing medical conditions. The algorithm starts with a relatively small learning set *MC* of medical conditions, composed by pairs , where is a technical term and a naïve term[[1]](#footnote-1), e.g.:

borborygmus stomach growling

emesis vomiting

First, the set *MC* is divided in three subsets and used for learning, refining and testing. The algorithm has five steps:

1. **Web mining step**: using , we extract from the Web sentence snippets including both terms, e.g. “*abdominal obesity, colloquially known as belly fat*”; “*chills are the frequent name for a feeling of coldness”*, “*sore throat, your doctor would call it pharyngitis*”, etc.
2. **Pattern generalization**: word patterns between medical conditions (e.g. *are the frequent name for*) as well as the medical conditions themselves (e.g. *feeling of coldness*) are generalized, using lexical, syntactic and semantic features. For example, p=”*is another word for*”, is generalized as *p’=be #ADJ word for”*, where “*is*” has been replaced by its lemma, and “*another”* by its part of speech (POS) #ADJ. To generalize, we also use semantic labels, such as BODYPART or DISCOMFORT. Discomfort words and body parts have been retrieved from publicly available Web resources[[2]](#footnote-2). Table 1 shows some of the most frequent patterns for medical conditions, Table 2 summarizes the rules to convert a lexical string into a generalized string.
3. **Clustering step**: patterns are clustered using an approach called *complete linkage* [10] and weighted according to the average distance between patterns. An example of cluster is:

{ *is known as, commonly known as the, often known as, known formally as, simply known as, known medically as,* etc. }and has 86 members.

1. **Reinforcement step**: using , we test the precision and recall of each pattern and adjust cluster weights;
2. **Testing phase**: Once a clustering has been learned, it is used to find unknown naïve terms. Extracted candidate terms are weighted according to the similarity of a supporting pattern with one of the clusters and to the number of patterns returning the same candidate. Notice that a new extracted pattern can be associated with a cluster (possibly with a very low similarity degree) also if neither its word string nor its generalized word string has been ever seen during the clustering step. A candidate term is accepted if its weight is over an experimentally tuned threshold $\beta.$ The algorithm performance is tested on the test set and all the steps are repeated for any possible permutation (six) of and . The final performance is computed as the average of the six experiments.
3. **Operational phase**: The best clustering result in step 5 is selected as the final model, and used to learn new mappings between technical and naïve terms.

| Sequence | Examples |
| --- | --- |
| NN | bilharzia, fainting, clenching, chickenpox |
| BODYPART NN | muscle weakness, skin writing, heart attack, hair fungus |
| JJ BODYPART | crooked tooth, stuffy nose, crooked back, dry mouth |
| inflammation of BODYPART | inflammation of the heart, inflammation of the liver, inflammation of the skin |

**Table 1.** Four most frequent generalized sequences for medical conditions (both *tt* and *nt*)

**Table 2**. String generalization rules for patterns (1) and medical conditions (2)

For training, refining and testing purposes we use a set *MC* of 193 pairs from Freebase. Notice that a term *t* in Freebase can have more than one *pt*, e.g.: *cephalalgia cephalodynia, headache, cranial pain,* therefore several pairs in *MC* can be generated from a single medical condition. Furthermore, the number of conditions for which there are alternatives in Freebase is currently about the size of our *MC,* and there is no distinction between technical and naïve terms: more simply, this resource collects sets of partner terms for every medical condition. Therefore, *tt* and *nt* have been paired manually.

To extract sentences we used the following web resources:

- Google snippets (up to the allowed query limits)
- Wikipedia
- BMC BioMed Central Corpus[[3]](#footnote-3)
- UKWaC British English web corpus[[4]](#footnote-4)

During each run of a testing phase, we take a from the dataset “playing the role” of and we try to extract from the previously listed web resources a set of correspondent partner terms, using the clusters and cluster weights learned in previous phases. We then compare them with the ground truth in . Let *TT* the set of technical terms in the test set and  the “true” set of naïve terms for each .

To compute performances, we use standard measures such as *precision*, *recall* and *F-measure*, as well as the *mean reciprocal rank (MRR)*, a measure that prizes true positives if they are top-ranked wrt the set of returned answers. *MRR* is defined as:

where is a true positive for retrieved by the algorithm (e.g. ), and is the position of in the list returned by the algorithm. We also compute the *coverage* as the ratio between the number of terms for which at least one answer is returned and those for which no alternative terms are found (over the threshold ).

The initial dataset was composed by 193 pairs from Freebase, partitioned in and . Since the test is repeated for any possible permutation of the three datasets, the performance is averaged over all the six experiments. In the six experiments, we collected a maximum of 860 and a minimum of 693 lexical patterns, from which we obtained maximum 637, minimum 471 generalized patterns and maximum 101, minimum 56 clusters.

The performance results are reported in Table 3. As expected, a higher threshold improves precision but reduces the coverage. Furthermore, since MRR is considerably higher than precision, we may conclude that true positives are likely to receive a higher score wrt false positives, which is a desired property.

|  | **Precision** | **MMR** | **Coverage** |
| --- | --- | --- | --- |
| 0 | 0.60 | 0.64 | 0.73 |
| 0.1 | 0.64 | 0.71 | 0.66 |
| 0.2 | 0.69 | 0.82 | 0.60 |

**Table 3**. Average system performance against golden-standard

Since often for a technical term there might be many naïve terms, and Freebase is far from being complete, two co-authors manually evaluated the extracted terms, regardless of the threshold. In Table 4 the Recall is computed considering the number of correct terms both above and below the threshold, rather than with reference to the golden standard. In the Table, *k-Fleiss* is the inter-annotator agreement[[5]](#footnote-5). The Table shows a higher precision, as expected, however there is quite a number of good terms below the threshold. The recall is lower than in Table 1 precisely because there is a higher number of naïve terms wrt the golden standard. In real cases, the better strategy is to use no threshold and ask a physician to mark the correct terms. Given a disease under surveillance, this manual step is simple and requires few minutes, while there would be no easy way for a clinician to imagine, without the help of a text mining tool, the variety of expressions used by patients.

|  | **Precision** | **Recall** | **F1** | **MMR** | **k-fleiss** |
| --- | --- | --- | --- | --- | --- |
| 0.2 | 0.76 | 0.49 | 0.59 | 0.74 | 0.53 |

**Table 4**. Manual Evaluation by domain experts

After the training phase, we selected the best performing clustering in the six experiments (namely, one with MRR=0,87) as the final model for extracting naïve medical language. We notice however that performances are not significantly variable and seem more related to the searched terms (i.e. whether they are more or less popular on the web) than to any of the clustering results.

1. In what follows, whenever a proposition applies to either a technical term or a naïve term, we use the notation *t* and *pt* (term and partner term) or *ct* (candidate partner term). [↑](#footnote-ref-1)
2. E.g. for discomfort: http://www.macmillandictionary.com/thesaurus-category/british/Physically-painful-and-describing-pain [↑](#footnote-ref-2)
3. http://www.biomedcentral.com/about/datamining [↑](#footnote-ref-3)
4. http://trac.sketchengine.co.uk/wiki/Corpora/UKWaC [↑](#footnote-ref-4)
5. http://en.wikipedia.org/wiki/Fleiss'_kappa#Interpretation [↑](#footnote-ref-5)
